# Influence of HLA Class I Haplotypes on HIV-1 Seroconversion and Disease Progression in Pumwani Sex Worker Cohort

**DOI:** 10.1371/journal.pone.0101475

**Published:** 2014-07-03

**Authors:** Raghavan Sampathkumar, Harold O. Peters, Lillian Mendoza, Thomas Bielawny, Elizabeth Ngugi, Joshua Kimani, Charles Wachihi, Francis A. Plummer, Ma Luo

**Affiliations:** 1 Department of Medical Microbiology, University of Manitoba, Winnipeg, Manitoba, Canada; 2 Department of Medical Microbiology, University of Nairobi, Nairobi, Kenya; 3 National Microbiology Laboratory, Public Health Agency of Canada, Winnipeg, Manitoba, Canada; University of Hawaii Manoa, United States of America

## Abstract

We examined the effect of HLA class I haplotypes on HIV-1 seroconversion and disease progression in the Pumwani sex worker cohort. This study included 595 HIV-1 positive patients and 176 HIV negative individuals. HLA-A, -B, and -C were typed to 4-digit resolution using sequence-based typing method. HLA class I haplotype frequencies were estimated using PyPop 32-0.6.0. The influence of haplotypes on time to seroconversion and CD4+ T cell decline to <200 cells/mm^3^ were analyzed by Kaplan-Meier analysis using SPSS 13.0. Before corrections for multiple comparisons, three 2-loci haplotypes were significantly associated with faster seroconversion, including A*23∶01-C*02∶02 (p = 0.014, log rank(LR) = 6.06, false-discovery rate (FDR) = 0.056), B*42∶01-C*17∶01 (p = 0.01, LR = 6.60, FDR = 0.08) and B*07∶02-C*07∶02 (p = 0.013, LR = 6.14, FDR = 0.069). Two A*74∶01 containing haplotypes, A*74∶01-B*15∶03 (p = 0.047, LR = 3.942, FDR = 0.068) and A*74∶01-B*15∶03-C*02∶02 (p = 0.045, LR = 4.01, FDR = 0.072) and B*14∶02-C*08∶02 (p = 0.021, LR = 5.36, FDR = 0.056) were associated with slower disease progression. Five haplotypes, including A*30∶02-B*45∶01 (p = 0.0008, LR = 11.183, FDR = 0.013), A*30∶02-C*16∶01 (p = 0.015, LR = 5.97, FDR = 0.048), B*53∶01-C*04∶01 (p = 0.010, LR = 6.61, FDR = 0.08), B*15∶10-C*03∶04 (p = 0.031, LR = 4.65, FDR = 0.062), and B*58∶01-C*03∶02 (p = 0.037, LR = 4.35, FDR = 0.066) were associated with faster progression to AIDS. After FDR corrections, only the associations of A*30∶02-B*45∶01 and A*30∶02-C*16∶01 with faster disease progression remained significant. Cox regression and deconstructed Kaplan-Meier survival analysis showed that the associations of haplotypes of A*23∶01-C*02∶02, B*07∶02-C*07∶02, A*74∶01-B*15∶03, A*74∶01-B*15∶03-C*02∶02, B*14∶02-C*08∶02 and B*58∶01-C*03∶02 with differential seroconversion or disease progression are due to the dominant effect of a single allele within the haplotypes. The true haplotype effect was observed with A*30∶02-B*45∶01, A*30∶02-C*16∶02, B*53∶01-C*04∶01 B*15∶10-C*03∶04, and B*42∶01-C*17∶01. In these cases, the presence of both alleles accelerated the disease progression or seroconversion than any of the single allele within the haplotypes. Our study showed that the true effects of HLA class I haplotypes on HIV seroconversion and disease progression exist and the associations of HLA class I haplotype can also be due to the dominant effect of a single allele within the haplotype.

## Introduction

HIV/AIDS continues to be a major public health concern, though decline in HIV prevalence has been reported for certain regions [1]. Women have been reported to bear half of the burden of HIV/AIDS [2]. The risk of female sex workers to be infected by HIV-1 is 13.5 fold greater than other women [3] due to heavy exposure to HIV through high risk sex work. The Pumwani sex worker cohort was established in 1985 and HIV-1 prevalence is about 70% [4]. Though the majority of women who were HIV-1 negative at cohort enrolment seroconverted within 3 years, there exists a small group of women remaining HIV uninfected despite heavy exposure [4], [5]. Some HIV-1 infected women maintained healthy CD4+ counts without anti-retroviral treatment for many years. Understanding of this natural immunity to HIV [6], [7] observed in the subgroup of sex workers in the Pumwani sex worker cohort could aid in designing an effective prophylactic and therapeutic vaccine.

Human leukocyte antigen (HLA) class I and II molecules present peptides to CD8+ and CD4+ T cells respectively [8]. HLA class I restricted HIV specific cytotoxic T lymphocytes (CTLs) play a predominant role in HIV control [9]–[11]. As HLA molecules initiate immune response via presentation of antigenic peptides to T cells, HLA-based HIV vaccine approach draws credence to combat HIV effectively [12]. Studying HLA allele and haplotype frequencies and dissecting individual and contextual role of alleles in influencing HIV/AIDS are critical to assess and advocate for specific multi-epitope vaccine constructs. Linkage disequilibrium (LD) is best exemplified in the HLA region [13] and this phenomenon demands careful investigation of indirect and interactive effects among alleles of different HLA genes in the context of disease outcome. HLA haplotypes are known to influence resistance or vulnerability to HIV-1 infection as well as development of opportunistic pathogen induced disease in HIV patients [14]–[18]. A study has shown among European Caucasians that HLA ancestral haplotype 8.1, 35.1, 44.2 and 35.2, 44.1, 57.1 were associated with rapid or slow progression to AIDS respectively [19]. A*24-B*40-Cw*03 and A*02-B*15-DRB1*12∶01 haplotypes were suggested to be protective against HIV infection in a Chinese population [20]. Association of haplotype HLA-A*23∶01-B*15∶03-C*02∶02 with increased mother-to-child HIV-1 transmission in a perinatal cohort from Nairobi has been reported [21]. Different populations exhibit varied immunogenetic profile, which is partly a reflection of responses to plethora of pathogens that has afflicted and continue to impact the specific population [22] and this warrants population specific characterization of host genetic profile and viral subtypes [12], [23]. We recently reported the association of HLA-A*01, C*06∶02, C*07∶01 with protection from seroconversion and A*74∶01, B*14, B*57∶03 with slower CD4+ T cell decline respectively in the Pumwani sex worker cohort [24]. Co-existence of favourable and unfavourable alleles from HLA class I loci as a haplotype in an individual could counteract, influencing the rate of seroconversion as well as disease progression. Hence we sought to address the nature of 2- and 3-loci haplotypes and their effects in HLA class I association with HIV-1 exposure outcome in Pumwani sex worker cohort.

## Methods

### Ethics Statement

Informed written consent was obtained from all study subjects and the Ethics Committee of the University of Manitoba as well as Ethics and Research Committee of Kenyatta National Hospital have approved this study.

### Study Population

The subjects involved in this study are enrollees of Pumwani sex worker Cohort, an open cohort with biannual follow-ups, established in 1985 in Nairobi, Kenya [5], [25]. Women in this cohort are routinely screened for HIV infection by serology and PCR amplification for *env*, *nef*, and *vif* genes. The present study includes 595 HIV positive patients (440 were HIV+ at the time of enrolment and 155 were seroconverters) and 176 HIV negative individuals. Among 176 HIV negative subjects, 118 were enrolled in the cohort before 1999 with an average follow-up time of 9.6±4.3 years. The demographic, clinical and behavioral characteristics of the HIV-negative and HIV-positive female sex workers included in the study have been published in a recent publication [24].

### HLA Class I typing

Human leukocyte antigen class I typing was performed as described earlier [24]. Briefly, DNA was isolated using QIAamp DNA Mini Kit and QIAgen EZ1 BioRobot (QIAGEN Inc, Mississauga, ON, Canada) and HLA-A, -B, and -C genes were amplified by PCR using gene specific primers. The purified PCR products were sequenced with BigDye cycle sequencing kits (Applied Biosystems, Foster City, CA) using sequence specific primers and analyzed with an ABI3100 Prism Genetic Analyzer. Allele specific primers were used to resolve ambiguous allele combinations. HLA-A, -B, and -C were typed to 4-digit resolution using CodonExpress, a software program developed based on a taxonomy-based sequencing analysis [26], [27].

### Estimation of class I haplotype frequencies and statistical analysis

HLA class I haplotype frequencies were estimated using PyPop version 32-0.6.0 [28] employing the expectation-maximization algorithm for haplotype estimation. HLA-A-B, B–C, A–C, A–B–C haplotype data was generated using 736, 724, 726 and 721 study subjects respectively. Haplotypes with frequency above 1% were coded with SPSS for seroconversion and disease progression analysis. All statistical analyses were conducted using SPSS 13.0. The associations of specific haplotypes with time to seroconversion (in a subset of HIV negative and seroconverters, n = 331) and CD4 decline to <200 cells/mm^3^ (in a subset of HIV positive and seroconverters, n = 384) were analyzed using Kaplan-Meier analysis. For seroconversion analysis, only women who were HIV-1 negative at enrolment and enrolled from 1985 to 2001 were included to ensure that the best defined biological phenotypes are being analyzed. A p value of <0.05 was considered statistically significant. For disease progression analysis, all the HIV-1 infected women with cohort entry CD4+ counts above 350/mm^3^ and women seroconverted after enrolment in the cohort were included in the analysis for CD4+ T-cell decline to below 200/mm^3^. Out of 440 HIV+ individuals with all three HLA class I genes typed, 211 HIV+ women whose entry CD4+ T cell counts <350/mm^3^ were excluded for this analysis. Thus, the analysis of CD4+ T cell decline included 229 HIV+ women. Because many women were HIV-1 infected at cohort entry, the inclusion of those with CD4+ counts above 350/mm^3^ increased sample size and the power of analysis and obtained results that is consistent with analysis of defined phenotype of rapid progressors (RP) (CD4+ T cell counts declined to below 400/mm^3^ within 3 years after seroconversion) and long term nonprogressors (LTNP) (maintaining CD4+ T cell counts above 400/mm^3^ for >7 years) with Fisher’s exact analysis [24]. All CD4 T cell count data from participants were prior to antiretroviral drug treatment initiation.

To examine whether the significant association of haplotypes with seroconversion or disease progression is due to individual allele or haplotype effect, we conducted **a**) Backward conditional Cox regression (Wald) analysis to determine the effect of individual alleles within the haplotype; **b**) deconstructed Kaplan-Meier survival analysis comparing the seroconversion or CD4+ decline of individuals with individual alleles of a haplotype with those who have all the constituent alleles of a specific haplotype. Because of the small sample size of the study population and high LD nature of the alleles that constitute the haplotypes, conducting analysis by completely excluding other allele(s) of a given haplotype in the meantime does not retain the statistical power. False-discovery rate (FDR) was used to adjust P value for multiple comparisons and the p values of 61 class I haplotypes with haplotype frequencies above 1% were used for FDR calculation. FDR was calculated using the FDR calculator in Excel file hosted at Rowett Research Institute website (http://www.rowett.ac.uk/~gwh/fdr.html).

## Results

### HLA-A-B, B-C, A-C and A-B-C haplotypes frequencies in the Pumwani sex worker cohort

Two and three loci haplotypes of the 3 classical HLA class I genes were identified using 771 women enrolled in Pumwani sex worker cohort. Haplotypes occurred at frequencies above 1% is provided in Table 1–4. There were 13 common A-B haplotypes, with the most prevalent ones being A*30∶01-B*42∶01 (3.6%), A*66∶01-B*58∶02 (2.5%) and A*02∶02-B*58∶02 (2.3%) (Table 1). Four B-C haplotypes, B*58∶02-C*06∶02, B*42∶01-C*17∶01, B*15∶03-C*02∶02 and B*53∶01-C*04∶01 were the most frequently observed haplotypes with frequencies >5.5% and phenotype frequencies >10% in this study population (Table 2). The high frequency haplotypes at A-C loci included A*30∶01-C*17∶01 (4.5%), A*66∶01-C*06∶02 (3.0%), A*02∶02-C*06∶02 (2.7%), A*01∶01-C*18∶01 (2.4%) and A*36∶01-C*04∶01 (2.1%) (Table 3). Although 3 loci haplotype analysis led to identification of 2814 haplotypes in this study population which is represented predominantly by highly homogenous Bantu speaking ethnicity [24], only 11 haplotypes occurred with >1% frequency (Table 4). A*30∶01-B*42∶01-C*17∶01 (3.8%), A*66∶01-B*58∶02-C*06∶02 (2.6%) and A*02∶02-B*58∶02-C*06∶02 (2.2%) are the most frequent haplotypes.

**Table 1:** The major HLA-A-B Haplotypes in Pumwani sex worker cohort.

HLA-A-B Haplotype	Frequency (%)(2n = 1458)	Phenotype Frequency (%)(n = 736)
A*30∶01-B*42∶01	53 (3.6)	54 (7.3)
A*66∶01-B*58∶02	37 (2.5)	39 (5.3)
A*02∶02-B*58∶02	34 (2.3)	35 (4.8)
A*36∶01-B*53∶01	28 (1.9)	27 (3.7)
A*01∶01-B*81∶01	26 (1.8)	30 (4.1)
A*02∶01-B*15∶03	25 (1.7)	30 (4.1)
A*74∶01-B*58∶02	23 (1.6)	33 (4.5)
A*74∶01-B*15∶03	22 (1.5)	21 (2.9)
A*68∶02-B*15∶10	21 (1.4)	25 (3.4)
A*30∶02-B*57∶03	17 (1.2)	30 (4.1)
A*03∶01-B*49∶01	15 (1.0)	17 (2.3)
A*68∶02-B*42∶01	15 (1.0)	20 (2.7)
A*30∶02-B*45∶01	15 (1.0)	19 (2.6)

Note: The ‘n’ represents the number of subjects studied; ‘2n’ indicates number of chromosomes; Numbers in parentheses represent percent frequency.

**Table 2:** The major HLA-B-C haplotypes in Pumwani sex worker cohort.

HLA-B-HLA-C Haplotype	Frequency (%)(2n = 1398)	Phenotype Frequency(%)(n = 724)
B*58∶02-C*06∶02	121 (8.7)	120 (16.6)
B*42∶01-C*17∶01	99 (7.1)	98 (13.5)
B*15∶03-C*02∶02	90 (6.4)	87 (12.0)
B*53∶01-C*04∶01	80 (5.7)	78 (10.8)
B*49∶01-C*07∶01	63 (4.5)	61 (8.4)
B*15∶10-C*03∶04	57 (4.1)	58 (8.0)
B*81∶01-C*18∶01	39 (2.8)	39 (5.4)
B*45∶01-C*16∶01	37 (2.6)	35 (4.8)
B*07∶02-C*07∶02	28 (2.0)	29 (4.0)
B*57∶02-C*18∶01	26 (1.9)	26 (3.6)
B*57∶03-C*07∶01	25 (1.8)	27 (3.7)
B*35∶01-C*04∶01	21 (1.5)	20 (2.8)
B*13∶02-C*06∶02	21 (1.5)	21 (2.9)
B*18∶01-C*07∶04	21 (1.5)	21 (2.9)
B*58∶01-C*07∶01	19 (1.4)	25 (3.5)
B*57∶03-C*18∶01	19 (1.4)	21 (2.9)
B*14∶06-C*08∶02	19 (1.4)	19 (2.6)
B*44∶03-C*04∶01	19 (1.4)	21 (2.9)
B*58∶01-C*06∶02	19 (1.4)	18 (2.5)
B*14∶02-C*08∶02	18 (1.3)	18 (2.5)
B*44∶15-C*04∶07	18 (1.3)	18 (2.5)
B*51∶01-C*16∶01	18 (1.3)	18 (2.5)
B*45∶01-C*06∶02	18 (1.3)	18 (2.5)
B*15∶03-C*04∶01	17 (1.2)	20 (2.8)
B*58∶01-C*03∶02	16 (1.1)	16 (2.2)

Note: The ‘n’ represents the number of subjects studied; ‘2n’ indicates number of chromosomes; Numbers in parentheses represent percent frequency.

**Table 3:** The major HLA-A-C haplotypes in Pumwani sex worker cohort.

HLA-A-HLA-C haplotype	Frequency (%) (2n = 1402)	Phenotype Frequency (%) (n = 726)
A*30∶01-C*17∶01	63 (4.5)	64 (8.8)
A*66∶01-C*06∶02	42 (3.0)	45 (6.2)
A*02∶02-C*06∶02	38 (2.7)	42 (5.8)
A*01∶01-C*18∶01	34 (2.4)	39 (5.4)
A*36∶01-C*04∶01	29 (2.1)	28 (3.9)
A*02∶01-C*16∶01	27 (1.9)	28 (3.9)
A*74∶01-C*06∶02	25 (1.8)	40 (5.5)
A*68∶02-C*03∶04	24 (1.7)	27 (3.7)
A*74∶01-C*02∶02	23 (1.6)	21 (2.9)
A*74∶01-C*07∶01	22 (1.6)	30 (4.1)
A*02∶01-C*04∶01	22 (1.6)	32 (4.4)
A*30∶02-C*18∶01	22 (1.6)	26 (3.6)
A*03∶01-C*07∶01	21 (1.5)	26 (3.6)
A*02∶01-C*02∶02	20 (1.4)	26 (3.6)
A*23∶01-C*02∶02	19 (1.4)	21 (2.9)
A*01∶01-C*07∶01	19 (1.4)	19 (2.6)
A*68∶02-C*07∶01	19 (1.4)	23 (3.2)
A*74∶01-C*04∶01	18 (1.3)	21 (2.9)
A*30∶02-C*04∶01	16 (1.1)	25 (3.4)
A*30∶02-C*16∶01	16 (1.1)	15 (2.1)
A*30∶02-C*07∶04	15 (1.1)	14 (1.9)
A*68∶02-C*07∶02	15 (1.1)	17 (2.3)
A*02∶01-C*07∶01	15 (1.1)	20 (2.8)

Note: The ‘n’ represents the number of subjects studied; ‘2n’ indicates number of chromosomes; Numbers in parentheses represent percent frequency.

**Table 4:** The major HLA-A-B-C haplotypes in Pumwani sex worker cohort.

HLA-A-B-C haplotype	Frequency(%)	PhenotypeFrequency
	(2n = 1388)	(n = 721)
A*30∶01-B*42∶01-C*17∶01	53 (3.8)	53 (7.4)
A*66∶01-B*58∶02-C*06∶02	36 (2.6)	36 (5.0)
A*02∶02-B*58∶02-C*06∶02	31 (2.2)	31 (4.3)
A*36∶01-B*53∶01-C*04∶01	27 (1.9)	26 (3.6)
A*74∶01-B*58∶02-C*06∶02	23 (1.7)	28 (3.9)
A*01∶01-B*81∶01-C*18∶01	22 (1.6)	23 (3.2)
A*74∶01-B*15∶03-C*02∶02	20 (1.4)	17 (2.4)
A*02∶01-B*15∶03-C*02∶02	20 (1.4)	22 (3.1)
A*68∶02-B*15∶10-C*03∶04	19 (1.4)	21 (2.9)
A*03∶01-B*49∶01-C*07∶01	15 (1.1)	16 (2.2)
A*74∶01-B*49∶01-C*07∶01	15 (1.1)	14 (1.9)

Note: The ‘n’ represents the number of subjects studied; ‘2n’ indicates number of chromosomes; Numbers in parentheses represent percent frequency.

### Haplotypes associated with faster seroconversion

**Figure 1:**
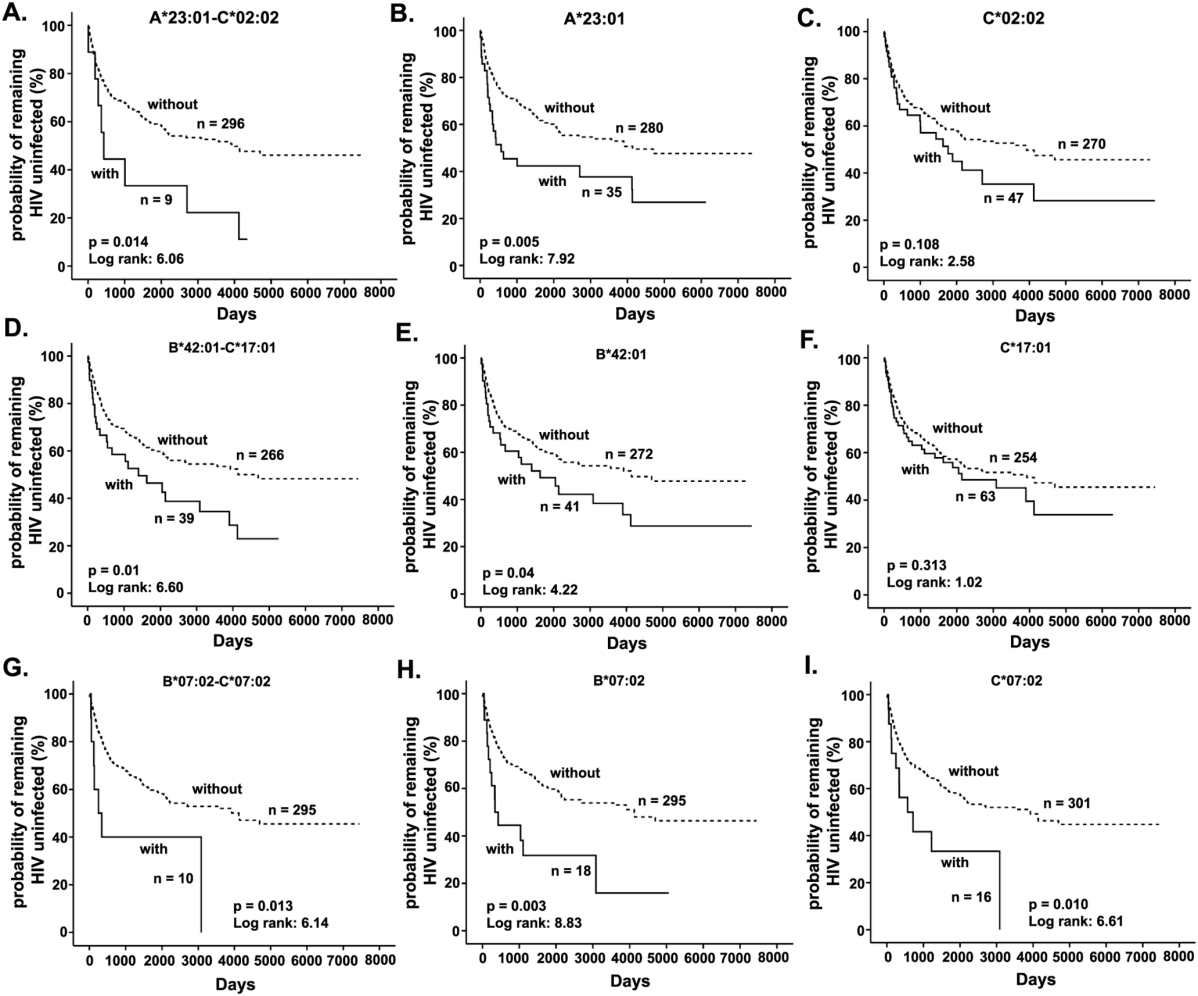
Kaplan-Meier plot of HLA class I haplotypes associated with rapid HIV-1 seroconversion, along with constituent alleles. (A) A*23∶01-C*02∶02; (B) A*23∶01; (C) C*02∶02; (D) B*42∶01-C*17∶01; (E) B*42∶01; (F) C*17∶01; (G) B*07∶02-C*07∶02; (H) B*07∶02; (I) C*07∶02. Solid line represents women with the specific haplotype or allele. Dashed line represents women without the specific haplotype or allele.

Survival analyses with 331 HIV negative and seroconverters identified the association of three 2 loci haplotypes, A*23∶01-C*02∶02 (p = 0.014, log rank (LR)  = 6.06), B*42∶01-C*17∶01 (p = 0.01, LR = 6.60) and B*07∶02-C*07∶02 (p = 0.013, LR = 6.14) with faster seroconversion (Table 5; Fig. 1A, 1D and 1G). The three haplotypes contained A*23∶01, B*42∶01 or B*07∶02. These alleles all have been identified to be associated with rapid seroconversion at the allele level in our previous study [24]. No enhanced associations (as indicated by stronger p value) of haplotypes A*23∶01-C*02∶02 and B*07∶02-C*07∶02 in faster seroconversion were observed when compared with the p value of survival analysis of individual alleles of each haplotype separately. However, stronger p value was observed in B*42∶01-C*17∶01 haplotype than each of the constitute alleles. Backward conditional Cox regression analysis showed that it is likely that A*23∶01 and B*07∶02 play a major role in the association of these haplotypes with faster seroconversion, while B*42∶01 and C*17∶01 together as a haplotype increased the rate of seroconversion. No haplotype associated with slower seroconversion has been observed. After FDR correction for multiple comparisons, the associations of these haplotypes with faster seroconversion were no longer significant (Table 5).

**Table 5:** HLA class I haplotypes associated with seroconversion and disease progression in Pumwani sex worker cohort.

Haplotype	Phenotype	Log Rank	P value	FDR	association
	Frequency (%)				
A*23∶01-C*02∶02	2.9	6.06	0.014	0.056	faster seroconversion
B*07∶02-C*07∶02	4	6.14	0.013	0.069	faster seroconversion
B*42∶01-C*17∶01	13.5	6.6	0.01	0.080	faster seroconversion
A*74∶01-B*15∶03	2.9	3.942	0.047	0.068	slower disease progression
A*74∶01-B*15∶03-C*02∶02	2.4	4.007	0.045	0.072	slower disease progression
B*14∶02-C*08∶02	2.5	5.364	0.021	0.056	slower disease progression
A*30∶02-B*45∶01	2.6	11.183	0.0008	**0.013**	faster disease progression
B*53∶01-C*04∶01	10.8	6.612	0.01	0.080	faster disease progression
B*15∶10-C*03∶04	8	4.648	0.031	0.062	faster disease progression
B*58∶01-C*03∶02	2.2	4.349	0.037	0.066	faster disease progression
A*30∶02-C*16∶01	2.1	5.966	0.015	**0.048**	faster disease progression

### Haplotypes associated with slower or faster disease progression

**Figure 2:**
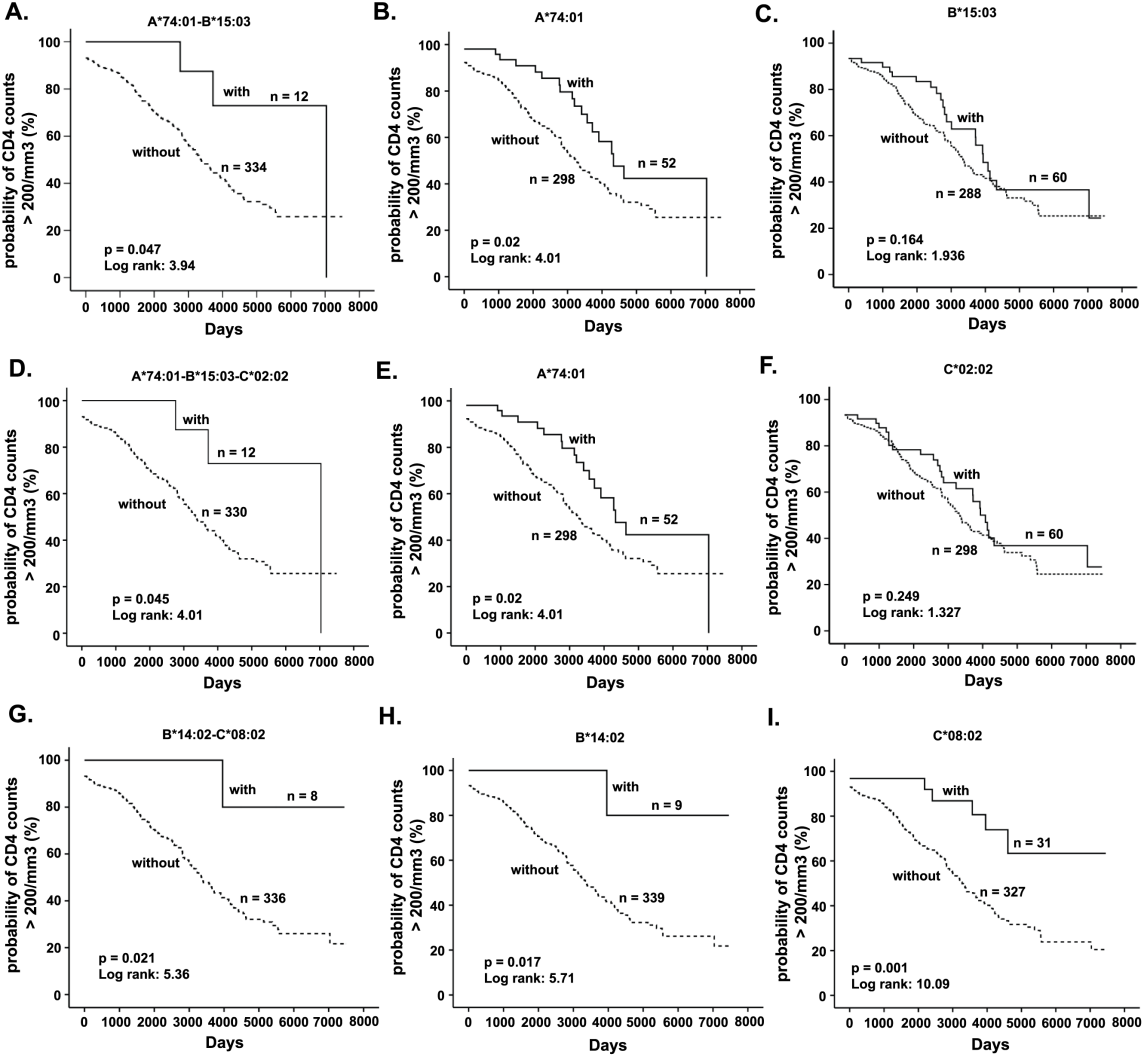
Kaplan-Meier plot of HLA class I haplotypes associated with slower HIV-1 disease progression, along with constituent alleles. (A) A*74∶01-B*15∶03; (B) A*74∶01; (C) B*15∶03; (D) A*74∶01-B*15∶03-C*02∶02; (E) A*74∶01; (F) C*02∶02; (G) B*14∶02-C*08∶02; (H) B*14∶02; (I) C*08∶02. Solid line represents women with the specific haplotype or allele. Dashed line represents women without the specific haplotype or allele.

**Figure 3:**
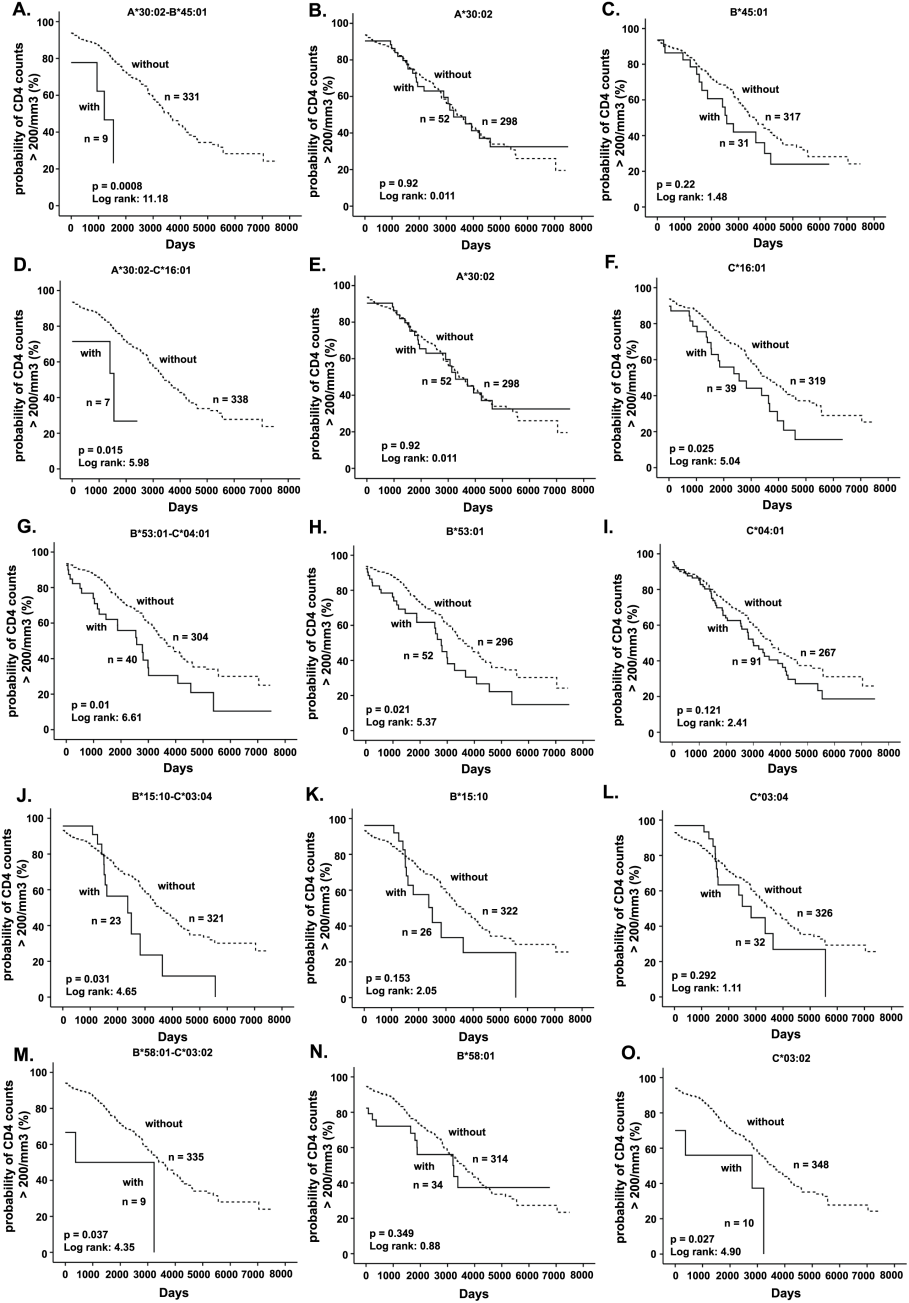
Kaplan-Meier plot of HLA class I haplotypes associated with rapid HIV-1 disease progression, along with constituent alleles. (A) A*30∶02-B*45∶01; (B) A*30∶02; (C) B*45∶01; (D) A*30∶02-C*16∶01; (E) A*30∶02; (F) C*16∶01; (G) B*53∶01-C*04∶01; (H) B*53∶01; (I) C*04∶01; (J) B*15∶10-C*03∶04; (K) B*15∶10; (L) C*03∶04; (M) B*58∶01-C*03∶02; (N) B*58∶01; (O) C*03∶02. Solid line represents women with the specific haplotype or allele. Dashed line represents women without the specific haplotype or allele.

Three A-B, B-C, or A-B-C haplotypes were associated with slower disease progression to CD4<200 cells/mm^3^ (Table 5) before correction for multiple comparisons. Patients possessing A*74∶01-B*15∶03 (p = 0.047, LR = 3.942), B*14∶02-C*08∶02 (p = 0.021, LR = 5.364), and A*74∶01-B*15∶03-C*02∶02 (p = 0.045, LR = 4.007) progressed to AIDS significantly slower than women without these haplotypes (Fig. 2). Women with A*74∶01-C*02∶02 (p = 0.089, LR = 2.984) and B*14∶06-C*08∶02 (p = 0.077, LR = 3.124) haplotypes had a trend towards slower CD4+ T cell decline. Whereas, women with either of the five haplotypes, A*30∶02-B*45∶01 (p = 0.0008, LR = 11.183), A*30∶02-C*16∶01 (p = 0.015, LR = 5.966), B*53∶01-C*04∶01 (p = 0.010, LR = 6.612), B*15∶10-C*03∶04 (p = 0.031, LR = 4.648), and B*58∶01-C*03∶02 (p = 0.037, LR = 4.349) progressed to AIDS more rapidly (Table 5; Fig. 3). A trend towards faster CD4+ T cell decline was observed in HIV+ patients with B*45∶01-C*06∶02 haplotype (p = 0.058, LR = 3.603). After FDR corrections for multiple comparisons, only the association of two haplotypes, A*30∶02-B*45∶01 and A*30∶02-C*16∶01, with faster disease progression remained significant (Table 5).

### Constituent alleles of the haplotype and their interactive effect on HIV-1 seroconversion and disease progression

Subsequent to the identification of haplotypes associated with either HIV-1 seroconversion or disease progression, we attempted to dissect whether the observed association is due to haplotype effect or the predominant effect by any of the constituent alleles using deconstructed Kaplan-Meier analysis and backward conditional Cox regression analysis. The analysis showed that the association of A*23∶01-C*02∶02 (p = 0.014, LR = 6.06) with rapid seroconversion could be attributed to A*23∶01 (p = 0.005, LR = 7.92) and not C*02∶02 (p = 0.108, LR = 2.58) (Table 6A and Fig. 1A–C). While C*17∶01 (p = 0.313, LR = 1.02) on its own was not associated with faster seroconversion, B*42∶01 (p = 0.04, LR = 4.22) did and the presence of both alleles enhanced the association of B*42∶01-C*17∶01 haplotype (p = 0.01, LR = 6.60) with faster seroconversion (Table 6B and Fig. 1D–F). In the case of B*07∶02-C*07∶02 (p = 0.013, LR = 6.14), although both alleles were associated with faster seroconversion, association of B*07∶02 (p = 0.003, LR = 8.83) is stronger than C*07∶02 (p = 0.010, LR = 6.61) by Kaplan Meier analysis (Fig. 1G–I) and by Cox regression analysis (Table 6C). The dominant effect of A*74∶01 (p = 0.02, LR = 4.01) in slowing CD4+ T cell decline was observed when it occurred with either B*15∶03 (p = 0.047, LR = 3.94) or B*15∶03 and C*02∶02 (p = 0.045, LR = 4.01) (Fig. 2A–F). Cox regression analysis also showed the dominant influence of A*74∶01 in these haplotypes (Table 7A and 7H). Similarly, in the case of the association of B*14∶02-C*08∶02 (p = 0.021, LR = 5.36) with slower progression, the effect of C*08∶02 appears to be dominant, as C*08∶02 (p = 0.001, LR = 10.09) alone showed stronger p value compared to B*14∶02 (p = 0.017, LR = 5.71) (Fig. 2G–I) and Cox regression analysis also demonstrated the dominant influence of C*08∶02 (Table 7F). In the case of B*58∶01-C*03∶02 (p = 0.037, LR = 4.35), rapid decline of CD4+ T cells could be attributed to C*03∶02 (p = 0.027; LR = 4.90) rather than B*58∶01 (p = 0.349, LR = 0.88) (Fig. 3M–O and Table 7G).

**Table 6:** Backward Cox Regression analysis of haplotype effect on seroconversion.

Variables in the Equation									
**A.**	A*23∶01-C*02∶02	**B**	**SE**	**Wald**	**df**	**Sig.**	**Exp(B)**	**95.0% CI for Exp(B)**	
								**Lower**	**Upper**
**Step 1**	A*23∶01-C*02∶02	−0.2310	0.5142	0.2018	1	0.6533	0.7937	0.2897	2.1745
	A*23∶01	−0.5061	0.2767	3.3460	1	0.0674	0.6028	0.3505	1.0368
	C*02∶02	−0.1872	0.2690	0.4845	1	0.4864	0.8292	0.4894	1.4049
**Step 2**	A*23∶01	−0.5704	0.2314	6.0773	1	0.0137	0.5653	0.3592	0.8897
	C*02∶02	−0.2482	0.2273	1.1930	1	0.2747	0.7802	0.4997	1.2180
**Step 3**	A*23∶01	−0.6096	0.2282	7.1378	1	0.0075	0.5436	0.3476	0.8501
**Variables in the Equation**									
**B.**	B*42∶01-C*17∶01	**B**	**SE**	**Wald**	**df**	**Sig.**	**Exp(B)**	**95.0% CI for Exp(B)**	
								**Lower**	**Upper**
**Step 1**	B*42∶01-C*17∶01	−8.7896	43.6369	0.0406	1	0.8404	0.0002	1.094E−41	2.121E+33
	B*42∶01	8.0026	43.6350	0.0336	1	0.8545	2988.8021	2.154E−34	4.147E+40
	C*17∶01	0.2749	0.3666	0.5626	1	0.4532	1.3165	0.6418	2.7004
**Step 2**	B*42∶01-C*17∶01	−0.9041	0.4278	4.4673	1	0.0346	0.4049	0.1751	0.9364
	B*17∶01	0.3713	0.3903	0.9053	1	0.3414	1.4497	0.6746	3.1152
**Step 3**	B*42∶01-C*17∶01	−0.5600	0.2210	6.4236	1	0.0113	0.5712	0.3704	0.8808
**Variables in the Equation**									
**C.**	B*07∶02-C*07∶02	**B**	**SE**	**Wald**	**df**	**Sig.**	**Exp(B)**	**95.0% CI for Exp(B)**	
								**Lower**	**Upper**
**Step 1**	B*07∶02-C*07∶02	0.4356	0.7551	0.3327	1	0.5641	1.5458	0.3519	6.7910
	B*07∶02	−0.7345	0.4185	3.0801	1	0.0793	0.4798	0.2112	1.0896
	C*07∶02	−0.6586	0.5103	1.6656	1	0.1968	0.5176	0.1904	1.4072
**Step 2**	B*07∶02	−0.5992	0.3689	2.6379	1	0.1043	0.5493	0.2665	1.1319
	C*07∶02	−0.4510	0.3987	1.2793	1	0.2580	0.6370	0.2916	1.3917
**Step 3**	B*07∶02	−0.8318	0.2918	8.1247	1	0.0044	0.4353	0.2457	0.7712

**Table 7:** Backward Cox Regression analysis of haplotype effect on disease progression.

Variables in the Equation									
**A.**	A*74∶01-B*15∶03	**B**	**SE**	**Wald**	**df**	**Sig.**	**Exp(B)**	**95.0% CI for Exp(B)**	
								**Lower**	**Upper**
**Step 1**	A*74∶01-B*15∶03	0.6331	0.6723	0.8870	1	0.3463	1.8835	0.5044	7.0340
	A*74∶01	0.4074	0.2734	2.2198	1	0.1362	1.5029	0.8794	2.5685
	B*15∶03	0.1235	0.2360	0.2741	1	0.6006	1.1315	0.7125	1.7968
**Step 2**	A*74∶01-B*15∶03	0.7508	0.6334	1.4050	1	0.2359	2.1186	0.6122	7.3313
	A*74∶01	0.3941	0.2724	2.0936	1	0.1479	1.4830	0.8696	2.5293
**Step 3**	A*74∶01	0.5650	0.2510	5.0681	1	0.0244	1.7594	1.0758	2.8773
**B.**	A*30∶02-B*45∶01	**B**	**SE**	**Wald**	**df**	**Sig.**	**Exp(B)**	**95.0% CI for Exp(B)**	
								**Lower**	**Upper**
**Step 1**	A*30∶02-B*45∶01	−1.5378	0.5964	6.6496	1	0.0099	0.2149	0.0668	0.6914
	A*30∶02	0.1848	0.2422	0.5824	1	0.4454	1.2030	0.7484	1.9338
	B*45∶01	−0.0410	0.3029	0.0183	1	0.8923	0.9598	0.5301	1.7378
**Step 2**	A*30∶02-B*45∶01	−1.5792	0.5123	9.5031	1	0.0021	0.2061	0.0755	0.5626
	A*30∶02	0.1884	0.2406	0.6132	1	0.4336	1.2074	0.7534	1.9350
**Step 3**	A*30∶02-B*45∶01	−1.4143	0.4659	9.2141	1	0.0024	0.2431	0.0975	0.6059
**C.**	A*30∶02-C*16∶01	**B**	**SE**	**Wald**	**df**	**Sig.**	**Exp(B)**	**95.0% CI for Exp(B)**	
								**Lower**	**Upper**
**Step 1**	A*30∶02-C*16∶01	−0.9691	0.6057	2.5597	1	0.1096	0.3794	0.1158	1.2437
	A*30∶02	0.1022	0.2379	0.1845	1	0.6676	1.1076	0.6948	1.7656
	C*16∶01	−0.3218	0.2478	1.6875	1	0.1939	0.7248	0.4460	1.1779
**Step 2**	A*30∶02-C*16∶01	−0.8658	0.5560	2.4251	1	0.1194	0.4207	0.1415	1.2509
	C*16∶01	−0.3379	0.2451	1.8999	1	0.1681	0.7133	0.4411	1.1532
**Step 3**	A*30∶02-C*16∶01	−1.1684	0.5125	5.1967	1	0.0226	0.3109	0.1138	0.8489
**D.**	B*53∶01-C*04∶01	**B**	**SE**	**Wald**	**df**	**Sig.**	**Exp(B)**	**95.0% CI for Exp(B)**	
								**Lower**	**Upper**
**Step 1**	B*53∶01-C*04∶01	−0.4123	0.5419	0.5789	1	0.4467	0.6621	0.2289	1.9151
	B*53∶01	−0.0749	0.4585	0.0267	1	0.8702	0.9278	0.3777	2.2789
	C*04∶01	−0.0799	0.2302	0.1203	1	0.7287	0.9232	0.5879	1.4498
**Step 2**	B*53∶01-C*04∶01	−0.4872	0.2895	2.8326	1	0.0924	0.6144	0.3484	1.0835
	B*04∶01	−0.0766	0.2293	0.1117	1	0.7382	0.9262	0.5909	1.4519
**Step 3**	B*53∶01-C*04∶01	−0.5511	0.2183	6.3757	1	0.0116	0.5763	0.3757	0.8840
**E.**	B*15∶10-C*03∶04	**B**	**SE**	**Wald**	**df**	**Sig.**	**Exp(B)**	**95.0% CI for Exp(B)**	
								**Lower**	**Upper**
**Step 1**	B*15∶10-C*03∶04	2.024	1.022	3.9220	1	0.0477	7.571	1.021	56.142
	B*15∶10	−0.635	0.710	0.798	1	0.3716	0.530	0.132	2.133
	C*03∶04	−0.974	0.710	1.8819	1	0.1701	0.378	0.094	1.518
**Step 2**	B*15∶10-C*03∶04	1.389	0.735	3.5749	1	0.0587	4.012	0.950	16.936
	C*03∶04	−0.958	0.710	1.8571	1	0.1730	0.380	0.095	1.528
**Step 3**	B*15∶10-C*03∶04	0.436	0.207	4.444	1	0.035	1.546	1.031	2.319
**F.**	B*14∶02-C*08∶02	**B**	**SE**	**Wald**	**df**	**Sig.**	**Exp(B)**	**95.0% CI for Exp(B)**	
								**Lower**	**Upper**
**Step 1**	B*14∶02-C*08∶02	1.0750	1.0959	0.9622	1	0.3266	2.9300	0.3420	25.1022
	B*14∶02			.	0^a^	.			
	C*08∶02	0.9574	0.4548	4.4314	1	0.0353	2.6050	1.0682	6.3525
**Step 3**	C*08∶02	1.2357	0.4168	8.7882	1	0.0030	3.4408	1.5200	7.7888
a. Degree of freedom reduced because of constant or linearly dependent covariates									
**G.**	B*58∶01-C*03∶02	**B**	**SE**	**Wald**	**df**	**Sig.**	**Exp(B)**	**95.0% CI for Exp(B)**	
								**Lower**	**Upper**
**Step 1**	B*58∶01-C*03∶02	−0.0798	1.1406	0.0049	1	0.9442	0.9233	0.0987	8.6350
	B*58∶01	−0.0759	0.3132	0.0588	1	0.8084	0.9269	0.5017	1.7123
	C*03∶02	−0.7667	1.0055	0.5813	1	0.4458	0.4646	0.0647	3.3340
**Step 2**	B*58∶01	−0.0821	0.2999	0.0749	1	0.7844	0.9212	0.5118	1.6582
	C*03∶02	−0.8281	0.4757	3.0302	1	0.0817	0.4369	0.1720	1.1099
**Step 3**	C*03∶02	−0.8894	0.4191	4.5037	1	0.0338	0.4109	0.1807	0.9343
**H.**	A*74∶01-B*15∶03-C*02∶02	**B**	**SE**	**Wald**	**df**	**Sig.**	**Exp(B)**	**95.0% CI for Exp(B)**	
								**Lower**	**Upper**
**Step 1**	A*74∶01-B*15∶03-C*02∶02	0.6435	0.6780	0.9010	1	0.3425	1.9032	0.5040	7.1870
	A*74∶01	0.3874	0.2738	2.0022	1	0.1571	1.4732	0.8614	2.5195
	B*15∶03	0.0946	0.3054	0.0960	1	0.7567	1.0992	0.6042	2.0000
	C*02∶02	0.0480	0.2901	0.0274	1	0.8686	1.0492	0.5942	1.8526
**Step 2**	A*74∶01-B*15∶03-C*02∶02	0.6580	0.6721	0.9582	1	0.3276	1.9308	0.5172	7.2088
	A*74∶01	0.3849	0.2734	1.9824	1	0.1591	1.4695	0.8599	2.5111
	B*15∶03	0.1268	0.2360	0.2889	1	0.5909	1.1352	0.7149	1.8027
**Step 3**	A*74∶01-B*15∶03-C*02∶02	0.7785	0.6333	1.5110	1	0.2190	2.1783	0.6295	7.5372
	A*74∶01	0.3714	0.2723	1.8598	1	0.1726	1.4498	0.8501	2.4724
**Step 4**	A*74∶01	0.5506	0.2510	4.8138	1	0.0282	1.7343	1.0605	2.8362

Notable haplotype effect was observed while dissecting four haplotypes associated with the rapid disease progression. While A*30∶02 (p = 0.92, LR = 0.011) and B*45∶01 (p = 0.22, LR = 1.48) alone did not demonstrate any detrimental effect, study subjects with A*30∶02-B*45∶01 haplotype progressed to AIDS rapidly (p = 0.0008, LR = 11.18) (Fig. 3A–C and Table 7B). The role of B*15∶10 (p = 0.153, LR = 2.05) and C*03∶04 (p = 0.292, LR = 1.11) in poor prognosis was noticed only when they occurred together (p = 0.031, LR = 4.65), demonstrating the interactive effects (Fig. 3J–L and Table 7E). As women with A*30∶02 (p = 0.92, LR = 0.011) did not progress to AIDS any faster and C*16∶01 (p = 0.025, LR = 5.04) positive women progressed to AIDS rapidly, the effect of A*30∶02-C*16∶01 haplotype (p = 0.015, LR = 5.98) was evident (Fig. 3D–F and Table 7C). B*53∶01-C*04∶01 is one of the most common haplotypes and occurred in 10.8% of the study population. The association of B*53∶01-C*04∶01 (p = 0.01, LR = 6.61) with rapid CD4+ decline is stronger than B*53∶01 (p = 0.021, LR = 5.37) or C*04∶01 (p = 0.121, LR = 2.41) alone (Fig. 3G–I and Table 7D). We also conducted analysis to identify individual allele effect of the four haplotypes, A*30∶02-B*45∶01, A*30∶02-C*16∶01, B*53∶01-C*04∶01 and B*15∶10-C*03∶04 on rapid CD4+ T cell decline by excluding individuals with the specified haplotypes. The analysis yielded similar results.

## Discussion

HLA class I alleles, especially those from HLA-A, -B and -C loci, have been repeatedly shown to exert profound influence over the observed inter-individual variability in vulnerability to infection and disease progression among HIV exposed individuals [29], [30]. Flores-Villanueva et al [19] reported that while HLA-B8 allele *per se* did not associate with rapid disease progression, its occurrence as part of complete ancestral haplotype 8.1 did show its association with faster progression. Contextual positioning of alleles and their potential interaction influences the outcome after HIV exposure and infection. We have recently reported favourable and detrimental associations of HLA Class I alleles with the rate of seroconversion and disease progression in Pumwani sex worker cohort [24]. As linkage disequilibrium is a hallmark of HLA loci [13], we sought to identify the common haplotypes and address whether HIV-1 seroconversion and disease progression could be under the influence of haplotype *per se* as well as individual alleles.

Our study has shown the significant influence of 11 2-loci and 3-loci haplotypes on HIV seroconversion and disease progression before correction for multiple comparisons. Faster HIV-1 seroconversion was observed in individuals possessing three 2-loci haplotypes, namely, A*23∶01-C*02∶02, B*42∶01-C*17∶01 and B*07∶02-C*07∶02. Three haplotypes (A*74∶01-B*15∶03, B*14∶02-C*08∶02, and A*74∶01-B*15∶03-C*02∶02) were associated with slower disease progression and 5 haplotypes (A*30∶02-B*45∶01, A*30∶02-C*16∶01, B*53∶01-C*04∶01, B*15∶10-C*03∶04, and B*58∶01-C*03∶02) were associated with rapid disease progression. Most of these haplotypes contained alleles previously identified to be associated with differential susceptibility to seroconversion or disease progression, such as A*23∶01, B*42∶01, B*07∶02, A*74∶01, B*14, B*15∶10, B*53∶01, and C*08∶02 [24]. Some of the results are consistent with findings of other African or North American populations. For example, A*23∶01-B*15∶03-C*02∶02 was associated with increased mother-to-child HIV-1 transmission in a perinatal cohort from Nairobi [21]. The association of B*53∶01-C*04∶01 with rapid disease progression is consistent with previous report of B*53-Cw*04 in faster disease progression in a North American population [9], [31].

We have not observed haplotype effect on slower seroconversion although several HLA class I alleles have been identified to be associated with slower seroconversion, such as A*01, C*06∶02 and C*07∶01 [24]. It is possible that fewer individuals with specific haplotypes reduced the power of detecting the association. Another explanation is that the adaptive immune response for preventing HIV-1 infection is diluted or neutralized by the non-protective or unfavorable immune response. HLA class I alleles present pathogenic peptides to CD8+ T cells to initiate CTL responses to destroy infected cells. Thus, the associations of HLA class I alleles and haplotypes with differential susceptibility to HIV-1 infection and disease progression could be attributed to their differential ability in HIV antigen presentations. In a previous study, we characterized HIV-1 Gag CD8+ T cell epitopes of A*01∶01 and B*07∶02, two HLA class I alleles associated with different outcomes of HIV-1 infection [32]. The study showed that for protection from HIV-1 infection, more might not be better. A*01∶01, an allele associated with slower seroconversion, has a very narrow epitope binding spectrum, while B*07∶02, an allele associated with rapid seroconversion, can present a broad spectrum of Gag epitopes and induce strong CD8+ T cell responses [32]. If the anti-HIV CD8+ T cell responses need to be focused on the conserved, vital part of HIV-1 to be protective, as seen in the Gag epitopes of A*01∶01, the increased spectrum of epitope recognition due to other HLA alleles constituting the haplotype will likely dilute or neutralize the protective effect of A*01∶01. These will need to be investigated and validated in future studies.

We used a deconstructed Kaplan-Meier analysis and Cox regression analysis to find out whether the associations of HLA class I haplotypes with differential susceptibility to HIV-1 infection and disease progression are the effect of haplotype or the constituent alleles. The analysis showed that the observed associations of these haplotypes with HIV seroconversion or disease progression can be attributed to either true haplotype effect or the dominant effect of a single allele within the haplotype. Haplotype effect has been observed in A*30∶02-B*45∶01, A*30∶02-C*16∶01, B*53∶01-C*04∶01 and B*15∶10-C*03∶04 for rapid disease progression, and B*42∶01-C*17∶01 for faster seroconversion. Although the associations of B*53∶01, B*15∶10 and B*42∶01 at the alleles level have been observed in our previous study [24], the co-existence of these alleles with C*04∶01, C*03∶04 and C*17∶01 respectively appears to enhance the association as indicated by the results of Cox regression analysis and the deconstructed Kaplan-Meier analysis. The association of two A*30∶02 containing haplotypes with rapid disease progression is new. The co-existence of A*30∶02 with either B*45∶01 or C*16∶01 appears to accelerate the rate of CD4+ T cell decline, while when A*30∶02 is alone, the effect is neutral. Because of the sample size of the study population, only the association of A*30∶02-B*45∶01 and A*30∶02-C*16∶01 with disease progression remained significant after correction for multiple comparisons. The dominant effect of A*23∶01, B*07∶02, A*74∶01, C*08∶02 and C*03∶02 has been observed in 6 haplotypes. Among them C*03∶02 is the only allele that has not been shown previously to be associated with differential disease progression. The association of A*74∶01 with slower disease progression to AIDS has also been reported in a South African population [33]–[35] and its influence on disease progression is also independent of its LD with B*57∶03 [35]. These results are consistent with our observation that A*74∶01 has a dominant effect in slower disease progression.

We have shown the correlative relationships of HLA class I haplotypes to both HIV-1 seroconversion and disease progression in Pumwani sex worker cohort. Haplotype analyses, including assessment of their constituent alleles, with two different survival end-points, time to seroconversion (in a subset of HIV negative and seroconverters) and CD4 decline (CD4<200 cells/mm^3^; in a subset of HIV positive and seroconverters) showed that haplotypes influencing seroconversion were not the ones impacting disease progression, reaffirming our observation in the allele level association study [24]. It indicates that immunological mechanisms influencing differential susceptibility to HIV infection are likely different from the ones impacting progression to AIDS.

The 6 alleles of three classical HLA class I genes restrict each individual’s CD8+ T cell responses to HIV-1 infection and disease progression. It is reasonable to assume that the outcome of HIV-1 infection and disease progression is influenced by CD8+ T cell responses restricted by all 6 class I alleles, each with varying effect. The alleles with dominant effect are most likely to be identified in association studies with phenotypically well-defined patient populations. Studying the effect of combinations of alleles or haplotypes on differential susceptibility to HIV-1 infection and disease progression is one step forward towards the understanding of the interactive effect of CD8+ T cell responses restricted by different HLA class I alleles. Although we cannot exclude the effect of non-HLA genes within the haplotypes, it appears that the associations of haplotypes with HIV-1 infection or disease progression in our study are either due to the dominant effect of a single allele or synergy of different alleles.

The sample size of phenotypically well-defined population is the major limiting factor in studying the effect of HLA class I haplotypes on HIV-1 infection and disease progression, given the extremely polymorphic nature of HLA class I genes. It is likely that some haplotype effects have been missed because of the sample size limitation of the study population. Despite this, our study has identified associations of HLA haplotypes with HIV seroconversion and disease progression, as well as validated some of previous findings in different populations. We hope that some of the results of our study can be confirmed by future studies of different patient populations.
